# Brain age trajectories and cognition after stroke in two longitudinal cohorts

**DOI:** 10.1093/braincomms/fcaf509

**Published:** 2026-01-20

**Authors:** Gisle Berg Helland, Håkon Ihle-Hansen, Anne Hege Aamodt, Esten H Leonardsen, Tobias Kaufmann, Brian Anthony B Enriquez, Mona K Beyer, Stein Andersson, Helle Stangeland, Hege Ihle-Hansen, Hanne F Harbo, Einar August Høgestøl, Guri Hagberg

**Affiliations:** Department of Neurology, Oslo University Hospital, 0424 Oslo, Norway; Department of Medicine, Bærum Hospital, Vestre Viken Hospital Trust, 3004, Drammen, Norway; Institute of Clinical Medicine, University of Oslo, 0318, Oslo, Norway; Department of Medicine, Bærum Hospital, Vestre Viken Hospital Trust, 3004, Drammen, Norway; Department of Neurology, Oslo University Hospital, 0424 Oslo, Norway; Department of Neuromedicine and Movement Science, The Norwegian University of Science and Technology, 7491, Trondheim, Norway; Primary Care and Mental Health, Faculty of Health and Life Sciences, University of Liverpool, L69 3GL, Liverpool, UK; Institute of Clinical Medicine, University of Oslo, 0318, Oslo, Norway; Department of Psychology, University of Oslo, 0373, Oslo, Norway; Institute of Clinical Medicine, University of Oslo, 0318, Oslo, Norway; Department of Psychiatry and Psychotherapy, Tübingen Center for Mental Health, University of Tübingen, 72076,Tübingen, Germany; German Center for Mental Health (DZPG), Partner Site Tübingen, 72076, Tübingen, Germany; Department of Neurology, Oslo University Hospital, 0424 Oslo, Norway; Institute of Clinical Medicine, University of Oslo, 0318, Oslo, Norway; Division of Radiology and Nuclear Medicine, Oslo University Hospital, 0424, Oslo, Norway; Department of Psychology, University of Oslo, 0373, Oslo, Norway; Department of Research and Innovation, Division of Clinical Neuroscience, Oslo University Hospital, 0424, Oslo, Norway; SHARE Centre for Resilience in Healthcare, Department of Quality and Health Technology, University of Stavanger, 4036, Stavanger, Norway; Department of Neurology, Oslo University Hospital, 0424 Oslo, Norway; Department of Medicine, Bærum Hospital, Vestre Viken Hospital Trust, 3004, Drammen, Norway; Department of Acute Medicine, Oslo University Hospital, 0424 Oslo, Norway; Institute of Health and Society, University of Oslo, 0450, Oslo, Norway; Department of Neurology, Oslo University Hospital, 0424 Oslo, Norway; Institute of Clinical Medicine, University of Oslo, 0318, Oslo, Norway; Department of Neurology, Oslo University Hospital, 0424 Oslo, Norway; Institute of Clinical Medicine, University of Oslo, 0318, Oslo, Norway; Department of Psychology, University of Oslo, 0373, Oslo, Norway; Department of Neurology, Oslo University Hospital, 0424 Oslo, Norway

**Keywords:** biomarker, stroke, follow-up studies, machine learning, cognition

## Abstract

Brain age is a promising neuroimaging biomarker, reflecting biological aging, but long-term trajectories and predictive value for cognitive outcomes post-stroke remains unclear. This study aimed to characterize brain aging trajectories over 8 years following a first-ever stroke and to evaluate the predictive value of brain age estimates for long-term cognitive outcomes. We analysed data from working-age (<65 years) ischaemic stroke patients with small- and medium-sized strokes (lesion volumes <70 ml), using two longitudinal stroke cohorts. T1-weighted MRI was acquired in the acute phase and at multiple time points up to 8 years post-stroke. Montreal cognitive assessment (MoCA) was assessed at follow-up sessions. Brain age was estimated using a state-of-the-art deep learning model. Brain-predicted age difference (Brain-PAD) was calculated as estimated brain age minus chronological age and corrected by regressing on age, age² and sex. Linear mixed-effects models examined Brain-PAD over multiple time points (whole-brain, ipsilesional and contralesional). Normalized brain volume was derived from FreeSurfer and included in the whole-brain analysis. Linear regression models evaluated whether brain age was associated with cognitive performance (MoCA) at long-term follow-up. We included 120 patients [*n* = 50 (42%) female, mean ± SD age at discharge was 54.9 ± 9 and National Institutes of Health Stroke Scale was 3.7 ± 6.4], with a mean follow-up of 3.4 ± 2.5 years. The mean MoCA score at follow-up was 24.7 ± 3.7. Brain-PAD increased significantly over time in the whole-brain analysis (*β* = 0.6/year, *P* < 0.01), indicating 60% acceleration in brain aging after stroke, with the association remaining significant after adjusting for normalized brain volume (*β* = 0.5/year, *P* < 0.01). Accelerated brain aging was observed in the ipsilesional hemisphere (*β* = 0.7/year, *P* < 0.01), but not the contralesional hemisphere (*β* = 0.3/year, *P* = 0.12). Higher brain age in the acute phase of stroke predicted lower MoCA scores at follow-up (*β* = −0.12, *P* < 0.05), whereas chronological age was not a significant predictor (*P* = 0.12). The association between brain age estimations and cognitive performance remained significant after adjusting for age, sex and education (*β* = −0.42, *P* < 0.01). In this longitudinal study, we found accelerated brain aging following stroke. Furthermore, brain age was associated with cognitive outcomes several years later, highlighting its potential as an early biomarker for long-term cognitive prognosis.

## Teaser

Stroke is considered an acute event. However, by utilizing novel machine-learning approaches, Berg Helland *et al*. demonstrate accelerated brain aging for up to 8 years following stroke, challenging this notion. The authors also show how brain age measured early after stroke can predict long-term cognitive outcomes.

## Graphical Abstract

**Graphical Abstract:**
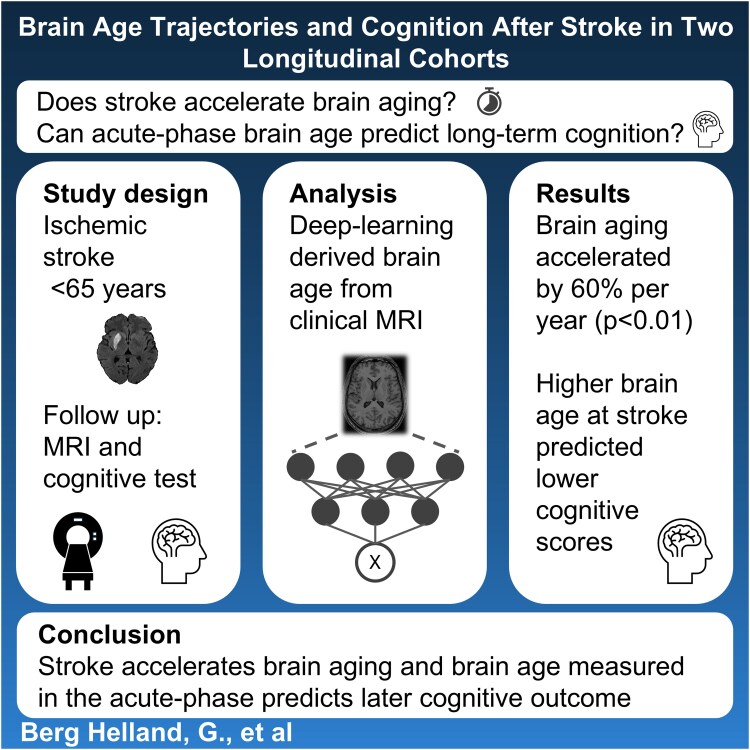

## Introduction

Chronological age is one of the strongest predictors of post-stroke outcomes.^1^ However, individuals of the same chronological age often show considerable variability in both health status and brain function. This variability has sparked growing interest in brain age, a neuroimaging-based biomarker that may offer additional patient-specific insights beyond chronological age.^2^ Brain age is estimated using machine-learning (ML) algorithms trained on MRI scans of healthy individuals, facilitating the assessment of natural aging processes as well as discoveries of deviations from these normal aging patterns.^3,4^

Although brain age models are trained on healthy brains, studies have shown that brain age estimations remain reliable over time, also for stroke patients.^5-7^ However, research on the trajectory of brain aging following stroke remains inconclusive. While one study found no evidence of accelerated aging,^6^ others have reported a significant association between stroke and accelerated brain aging in the sub-acute phase, emphasizing the need for more longitudinal studies.^8^

Beyond understanding brain aging trajectories after stroke, there is a need to assess the clinical utility of estimating brain age after stroke. Post-stroke cognitive impairment is common, affecting up to half of all stroke survivors,^9^ and brain age may provide additional information beyond chronological age in the prediction of cognitive decline following stroke. However, to date, few studies have examined the relationship between brain age and long-term cognitive outcomes after stroke, even though existing evidence suggests that having an older-appearing brain may be associated with increased risk of post-stroke neurocognitive disorder.^10^

Previous brain age studies in stroke populations have relied on brain age models that require extensive MRI pre-processing pipelines and converting images into tabular data before running a ML model.^7,11,12^ However, new technology using deep learning (DL), allows models to process raw MRI images in just a few minutes with robust performance,^13^ although this approach remains to be tested in stroke populations.

Accordingly, in this study, we aim to characterize longitudinal brain age trajectories after stroke and investigate the association between acute-phase brain age estimations and long-term cognitive outcome using a publicly available DL model for brain age estimation.

## Materials and methods

### Study design and participants

This study included patients younger than 65 years at the time of hospital admission with small to medium-sized strokes (lesion volume <70 ml),^14^ from two stroke cohorts. The cut-off was based on previous brain aging studies, where large stroke lesions present inherent challenges for MRI post-processing,^7,15^ and was later confirmed by our own visual quality control. The first cohort included all patients admitted to the Stroke Unit at Bærum Hospital, Vestre Viken, between 2007 and 2008, as part of the Cognition after Stroke Trial (CAST), including cases of ischaemic stroke, haemorrhagic stroke and transient ischaemic attack (TIA).^16^ Patients were followed at multiple time points, with the last follow-up between 2013 and 2016.^17^ For the present study, we included only those with a diagnosis of ischaemic stroke, excluding patients with haemorrhagic stroke or TIA.

The second cohort consisted of patients from the *Oslo Acute Revascularization Registry* (OSCAR), which included individuals with acute large-vessel occlusion admitted for endovascular treatment to the Stroke Unit at Rikshospitalet, Oslo University Hospital.^18,19^ For this study, we included patients admitted between 2017 and May 2019. Participants who were under 65 years of age at the time of stroke were invited for long-term follow-up between 2021 and 2022.

### Clinical variables

Clinical and cognitive assessments were performed at the study follow-up visits. Stroke severity was evaluated using the National Institutes of Health Stroke Scale (NIHSS),^20^ which was administered at each time point. Cognitive function was assessed with the MoCA test version 7.1,^21^ measured during the 2013–16 follow-up in the CAST cohort and during the 2021–22 follow-up in the OSCAR cohort. Raw (i.e. not adding an extra point to those with ≤12 years of education) scores were used in the analysis, as information on education was missing for some patients. Educational status was recorded at baseline in the CAST cohort and retrospectively in the OSCAR cohort, at the 2021–22 follow-up. Education was categorized as high (>12 years of formal schooling) or low (≤12 years). Stroke lesion volumes were segmented using diffusion-weighted imaging (DWI) or fluid-attenuated inversion recovery (FLAIR) images, and details of the lesion segmentation procedure used to derive stroke lesion volumes are provided in the Supplementary material.

### Neuroimaging data acquisition and pre-processing

In the CAST cohort, all MRI scans were acquired using the same scanner across time points: a Philips Intera 1.5 Tesla (T) system. A 3D T1-weighted (T1w) fast field echo sequence was used at follow-up time points in 2008–09 and 2013–16. Acute-phase scans (acquired in 2007–08) were performed using a 2D T1w Spin Echo sequence and were excluded from the main brain age analysis, as well as analyses on early phase images and long-term cognitive outcomes, due to significant differences in image quality and dimensionality.

In the OSCAR cohort, T1w scans were acquired across four different Siemens scanners: one Avanto 1.5T, two Aera 1.5T systems and one Skyra 3T system. All scanners employed a 3D T1w magnetization prepared rapid acquisition gradient echo (MPRAGE) sequence. Detailed sequence parameters for each scanner, as well as number of scans performed at each time point, are provided in Supplementary Tables 1 and Supplementary Table 2, respectively. All patients (*n* = 120) were scanned at least once during the study period.

All MRI data were extracted from hospital servers as de-identified digital imaging and communications in medicine (DICOM) files. The DICOM files were then converted to Neuroimaging Informatics Technology Initiative (NIfTI) format using dcm2niix and organized according to the brain imaging data structure (BIDS) format.^22^ The resulting BIDS-formatted NIfTI files were used for all subsequent analyses.

### Brain age analysis

#### Brain age estimation

Brain age was estimated using a publicly available DL model pre-trained on healthy participants (*n* = 34 285) from 21 publicly available, non-overlapping datasets, with an age range spanning from 3–95 years (https://github.com/estenhl/pyment-public).^23^ Detailed processing steps within the DL model framework are found in the Supplementary material. The model, trained to recognize normal aging, outputs an individual estimated brain age for an MRI scan.

Brain age was estimated using each patients T1w image (whole-brain analysis) at each time point. Brain age estimations were not available for 10 patients (8%) after initial MRI processing, and subsequent visual inspection revealed that the corresponding MRI images were affected by artefacts and/or of poor quality.

In addition to the estimation of whole-brain aging effects of ischaemic stroke, we aimed to investigate potential differences in brain aging between the ipsilesional (lesioned) and contralesional (non-lesioned) hemispheres. Since the DL model requires a full brain volume MRI as input, we generated hemisphere-specific inputs by mirroring T1-weighted brain MRI scans along the left-right axis using a custom Python script, detailed in Supplementary material. The ipsilesional and contralesional volumes were then processed with the DL model to generate hemisphere-specific brain age estimations. Patients with posterior infarcts were excluded from this sub-analysis, due to cases with lack of, or bilaterally, lesioned hemisphere. A schematic overview of the MRI pipeline and the DL model is provided in Supplementary Fig. 1.

### Brain-predicted age difference

The difference between the estimated brain age and the person's chronological age, known as the brain-predicted age difference (Brain-PAD), indicates whether the brain is estimated to be younger, older, or consistent with the person's actual age. This is sometimes calculated as Brain-PAD = brain age—chronological age.^7^ However, due to the known tendency of ML-based brain age models to regress towards the mean, meaning they systematically underestimate the age of older individuals and overestimate that of younger individuals,^24^ bias correction is usually applied.^25^ We corrected for age bias by regressing this raw Brain-PAD value on chronological age, age^2^ and sex:


$$\mathrm{Brain}\text{-}\mathrm{PA}D_{i}=\beta_{0}+\beta_{1}\times \mathrm{Ag}e_{i}+\beta_{2}{\times \mathrm{Age}}_{i}^{2}+\beta_{3}\times \mathrm{Se}x_{i}+\varepsilon_{i}$$

**Figure 1:**
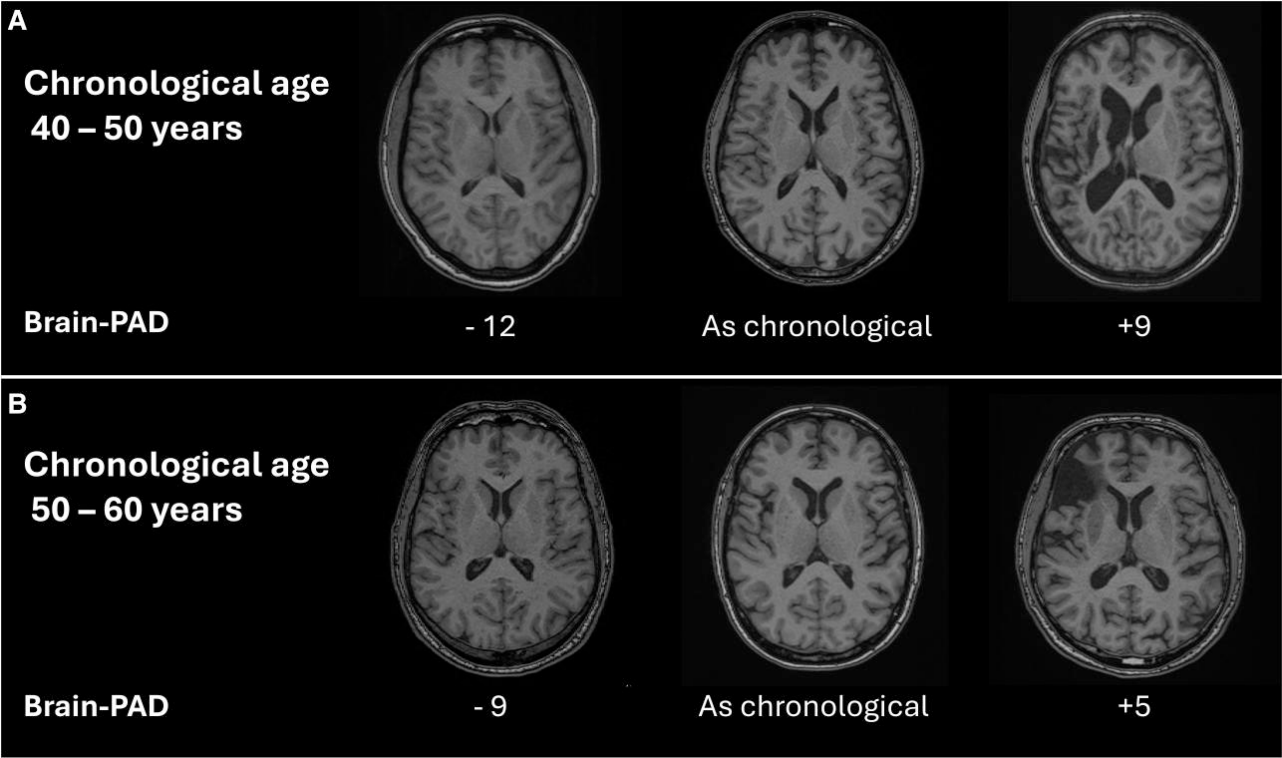
**Examples of older and younger appearing brains.** Examples of Brain-PAD alongside the corresponding input T1-weighted MRI images. Both panel (**A**) and panel (**B**) illustrate brains appearing younger-, older- or in line with chronological age. Panel (**A**) shows three examples from individuals aged 40–50 years, while Panel (**B**) shows three examples from individuals aged 50–60 years. The Brain-PAD values were corrected for age and sex bias. Exact ages are withheld to ensure de-identification.

The age-bias correction was performed separately for each estimate, using all available data points from that specific model. The residuals from this model (*ε_i_*), representing age- and sex-corrected Brain-PAD values, were used as the primary outcome for longitudinal brain age analysis. A visualization of the residual (constituting the corrected Brain-PAD) and raw Brain-PAD is provided in Supplementary Fig. 2 and Supplementary Fig. 3. Examples of younger, older and chronologically looking brains are shown in Fig. 1, with corresponding T1w images.

### Supporting measures for brain age analysis

To substantiate our findings, we performed exploratory analyses using FreeSurfer-derived outputs. The processing pipeline of these automatic segmentation volumes is detailed in the Supplementary material. First, we calculated FreeSurfer-derived normalized brain volume (global brain volume without ventricles divided by intracranial volume, expressed as a percentage) to examine whether brain aging was linked to brain volume across timepoints,^26^ and whether changes in Brain-PAD over time were associated with brain atrophy. Second, to allow for comparison with earlier post-stroke brain age studies, which have all used tabular data, we estimated brain age using a separate ML-model eXtreme Gradient Boosting (XGBoost) trained on basic FreeSurfer-derived and subcortical features.^27^

We also performed an exploratory analysis, where all patient T1w MRIs (including legacy scans acquired with 2D T1w sequences) were transformed using SynthSR^28^ provided by FreeSurfer, in an attempt to convert clinical images into standardized, lesion-filled, MPRAGE 3D T1w images.^29^ See Supplementary material for details on XGBoost and SynthSR-based processing, respectively.

### Statistical analysis

All statistical analyses were performed using R software (version 4.4.2).^30^ We report the number of patients assessed at each follow-up. Categorical variables are presented as counts and percentages, while continuous variables are summarized using means, standard deviations and range. Segmented lesion volume was log-transformed to reduce skewness. Statistical comparisons of admission and discharge variables between participants who adhered to the study and those lost to follow-up were first based on tests of assumption. Normality was assessed using the Shapiro–Wilk test and homogeneity of variance with Levene’s test. If assumptions for parametric testing were violated, the Wilcoxon rank-sum test was applied. For categorical variables, the χ^2^ test was used when all cell counts were five or more.

### Longitudinal brain age analysis

To account for repeated assessments within individuals, linear mixed-effects (LME) models were used with Brain-PAD as the outcome variable and subject ID as a random effect. All other variables were included as fixed effects. The main independent variable of interest was time since stroke, where a significant time effect would indicate Brain-PAD changes over time, with a positive effect reflecting accelerated brain aging. Secondary independent variables included lesion volume, to assess whether damaged tissue influences brain age estimations; total brain volume, to evaluate whether brain aging could be explained by volumetric loss alone and education, as a potentially modifiable factor associated with brain aging. Vascular territory was included to account for regional stroke-related effects on aging. Finally, sex and chronological age were examined in separate univariable models to confirm that their effects had been effectively regressed out during the brain age correction procedure.

To investigate the association between brain volume change over time and brain aging, we calculated within-subject changes in normalized brain volume and Brain-PAD between consecutive sessions and assessed their relationship using Pearson correlation.

### Brain age and cognition

We used linear regression models to investigate associations between brain age in the acute phase of stroke and long-term post-stroke cognitive performance, measured with the MoCA. We included chronological age and sex as covariates in the model rather than pre-correcting brain age estimates directly, as such correction could remove meaningful shared variance and increase the risk of false negatives. To validate this decision, we compared adjusted and unadjusted models and found that while correcting for covariates increased model complexity, it did not significantly improve model fit (ANOVA, *P* = 0.07). Therefore, the final models included uncorrected brain age alongside chronological age and sex as covariates. Education, lesion volume and vascular territory were also evaluated in separate univariable models to assess their individual associations with cognitive outcome and explore potential confounding effects.

### Multivariable models

Multivariable models were made for both LME and linear regression, and variables were included in the multivariable models if they had a *P*-value < 0.20 in univariable analysis. This threshold was chosen to ensure that variables with potential effects were considered, even if they did not reach significance in univariable models.

#### Ethical approval

This study was conducted in accordance with the Declaration of Helsinki,^31^ with ethical approval obtained from the Regional Committee for Medical and Health Research Ethics in Southeast Norway (REK). Approval was granted separately for each cohort: CAST (REK ID: 2013/1829) and OSCAR (REK ID: 152864).

## Results

### Participant characteristics

**Figure 2:**
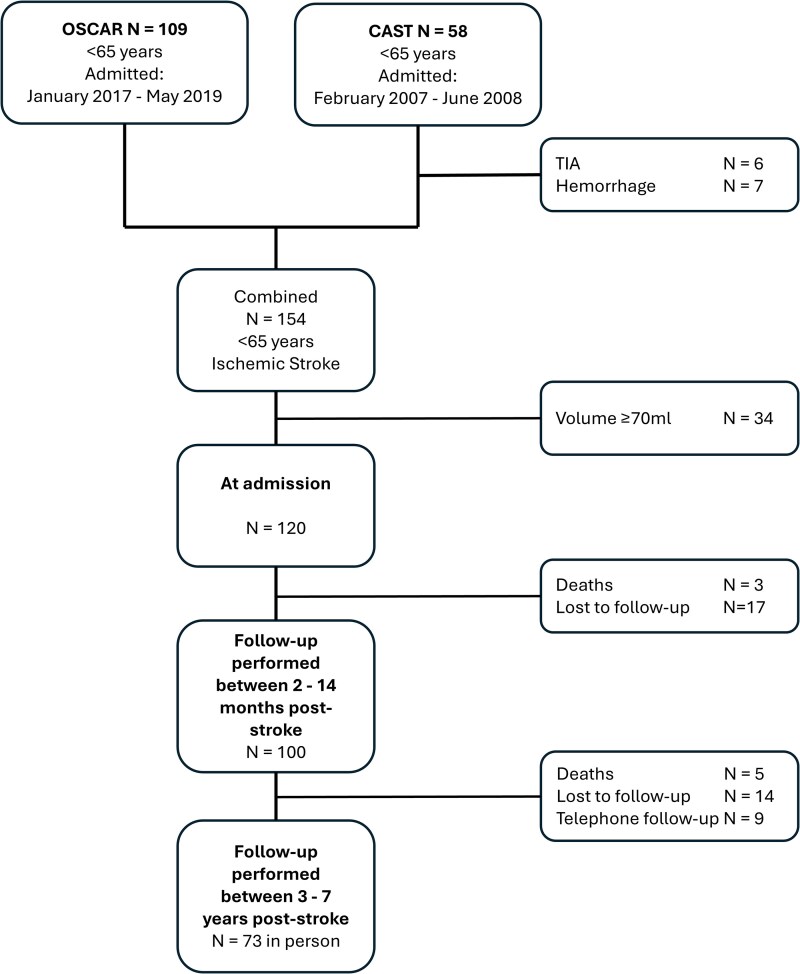
**Study overview.** Flowchart visualizing patients in each of the two cohorts. The OSCAR and CAST, and subsequent inclusion, exclusion and loss to follow-up.

A total of 120 patients from the two stroke cohorts were included and followed for a mean ± SD of 3.4 ± 2.5 years. At admission, the mean age was 54.9 ± 9 years, 42% (*n* = 50) were female, the mean NIHSS score was 9 ± 8 and the mean lesion volume was 15 ± 17 ml, as shown in Table 1. At final follow-up (4.8 ± 1.7, range 2.8–8.7 years), 73 patients (61%) attended in person. See the flowchart in Fig. 2 and Supplementary Table 3 and Supplementary Table 4 for details on follow-up. There were no significant differences between those who adhered to the CAST cohort and those who dropped out. In the OSCAR cohort, however, dropouts at the ∼3-month follow-up were older (*P* = <0.05), while dropouts at the ∼3-year follow-up had higher admission and discharge NIHSS scores (*P* = <0.05), indicating more severe strokes.

**Table 1:** Demographic and clinical characteristics at admission, discharge and follow-up

Characteristics	*N* = 120
Admission	
Age, in years, mean (SD)	55 (9)
Sex, female *n* (%)	50 (42%)
NIHSS, mean ± SD; [range]	9 ± 8 [0–39]
Education, higher (%)^a^	24 (40%)
Arterial territory^b^	
Left media	47 (39%)
Right media	39 (33%)
Posterior	26 (22%)
Discharge	
Number of patients	120
Time since stroke, days, mean ± SD; [range]	4 ± 6 [0–28]
NIHSS, mean ± SD; [range]^c^	4 ± 6 [0–39]
Lesion volume, mean ± SD; [range]	15 ± 17 [0–68]
Follow-up^d^	
Number of patients	100
Time since stroke, months, mean ± SD; [range]^e^	6.8 ± 4.3 [2–14]
NIHSS, mean ± SD; [range]	1 ± 2 [0–12]
Follow-up^f^	
Number of patients	73
Time since stroke, months, mean ± SD; [range]^g^	58 ± 20 [33–104]
NIHSS, mean ± SD; [range]	1 ± 2 [0–7]
MoCA, mean ± SD; [range]^h^	24.7 ± 3.7 [14–30]

NIHSS, National Institutes of Health Stroke Scale; MoCA, Montreal Cognitive Assessment.

Missing: ^a^11, ^b^8, ^c^1, ^d^follow-up indicates the patients’ first outpatient assessment, ^e^3, ^f^follow-up indicates the patients’ final outpatient assessment, ^g^4, and ^h^1.

Brain age estimates derived from the DL model, was significantly correlated to chronological age (*r* = 0.84, *P* < 0.001) and the model yielded a mean absolute error of 4.5 years for the whole-brain analysis, visualized in Supplementary Fig. 3.

### Longitudinal brain age results

**Figure 3:**
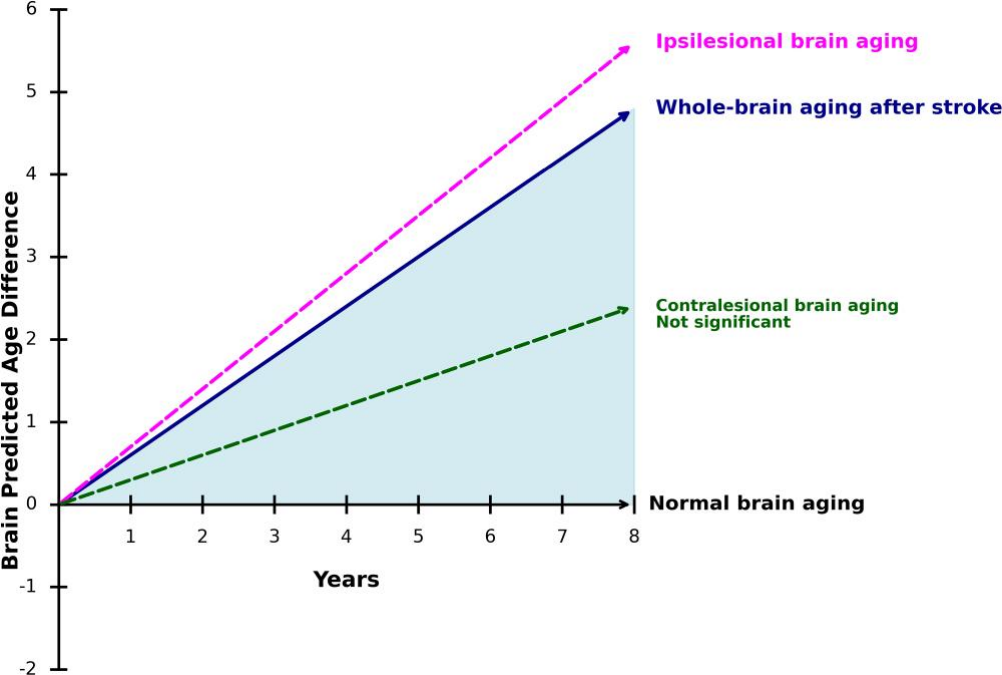
**Accelerated brain aging after stroke.** Illustration of the rate of accelerated brain aging after stroke, based on Brain-PAD, estimated with linear mixed-effect models. Whole-brain analysis showed a 60% increase (*P* < 0.01, *N* = 209 T1w scans), the ipsilesional hemisphere a 70% increase (*P* < 0.01, *N* = 155 T1w scans), and the contralesional hemisphere a 30% increase (*P* = 0.12, *N* = 155 T1w scans).

Results from the LME models showed a significant positive association between corrected Brain-PAD and time since stroke (*β* = 0.6, *P* < 0.01), as seen in Table 2, which corresponds to 60% accelerated brain aging compared with chronological aging. Significant accelerated brain aging was observed in the ipsilesional side (*β* = 0.7, *P* < 0.01), but not in the contralesional side (*β* = 0.3, *P* = 0.12). Corresponding brain aging trajectories over time are illustrated in Fig. 3.

**Table 2:** Linear mixed-effect models, association between brain-predicted age difference Brain-PAD and variables post-stroke

	Univariable		Multivariable	
	Effect size (CI 95%)	*P*-value	Effect size (CI 95%)	*P*-value
Whole-brain estimations				
Sex, female	0.01(−1.8–1.8)	0.99		
Age	0.03 (−0.1–0.1)	0.6		
Time since stroke, years	**0.6** (**0.3–0.9)**	**<0**.**01**	**0.4** (**0.1–0.7)**	**<0**.**05**
Lesion volume, log	0.4 (−0.3–1.2)	0.26		
Education, lower	−0.3 (−2.6–2.0)	0.82		
Territory				
Right media	−1.1 (−3.1–0.9)	0.27	−0.3 (−2.4–1.8)	0.79
Posterior	−1.6 (−3.8–0.7)	0.18	−1.0 (−3.4–1.4)	0.42
Brain volume (%), normalized	**−0.6** (**−0.8-−0.4)**	**<0**.**01**	**−0.5** (**−0.6-−0.3)**	**<0**.**01**
Ipsilesional estimations				
Sex, female	−0.03 (−2.2–2.1)	0.99		
Age	0.01 (−0.11–0.14)	0.82		
Time since stroke, years	**0.7** (**0.3–1.1)**	**<0**.**01**	**0.7** (**0.3–1.1)**	**<0**.**01**
Lesion volume, log	−0.07 (−1.0–0.9)	0.89		
Education, lower	−1.7 (−4.1–0.7)	0.17		
Territory, right media	−1.3 (−3.4–0.8)	0.23		
Contralesional estimations				
Sex, female	0.1 (−2.2–2.4)	0.94		
Age	0.00 (−0.12–0.13)	0.99		
Time since stroke, years	0.3 (−0.1–0.6)	0.13	0.3 (−0.1–0.6)	0.13
Lesion volume, log	−0.4 (−1.4–0.6)	0.39		
Education, lower	−0.05 (−3.0–2.9)	0.97		
Territory, right media	−1.2 (−3.5–1.1)	0.31		

Bold values indicate statistical significance (*P* < 0.05).

Normalized brain volume was also significantly associated with brain aging estimated by the DL model on the native T1w images, where a 1% loss in normalized brain volume was associated with a 0.6-year increase in corrected Brain-PAD (*P* < 0.01). Notably, both normalized brain volume and time since stroke remained significant in the multivariable model (*β* = –0.5, *P* < 0.01 and *β* = 0.4, *P* < 0.05). Furthermore within-subject changes in brain volume over time were significantly negatively correlated with changes in corrected brain-PAD (*r* = −0.62, *P* < 0.01).

Results from the exploratory analyses, including the XGBoost brain age model and SynthSR processed images, are presented in Supplementary Table 5.

### Association of acute-phase brain age estimation and long-term cognitive performance

**Figure 4:**
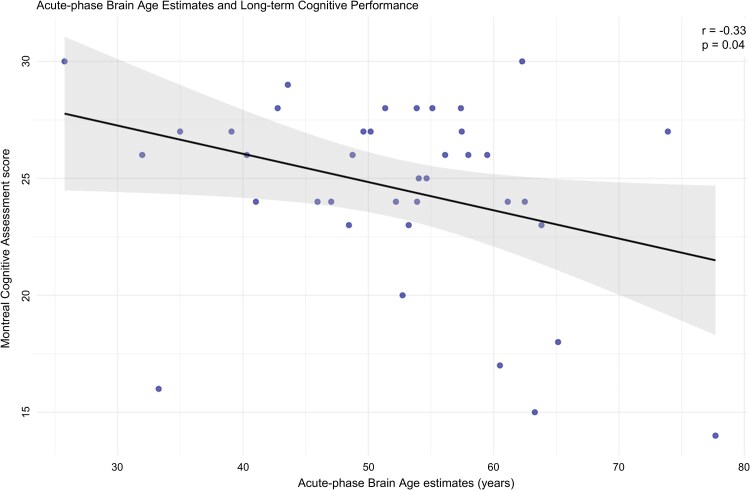
**Brain age and cognition.** Scatterplot showing DL-based brain age estimates in the acute phase after stroke versus MoCA scores 3 years later. The line represents the fitted regression together with a 95% confidence interval, and dots indicate individual data points (*N* = 38). *r*: Pearson correlation coefficient.

The significant correlation (*r* = −0.33, *P* < 0.05) between long-term cognitive performance, measured with the MoCA and acute-phase brain age estimates is visualized in Fig. 4. Linear regression models revealed a significant association between increased acute-phase brain age estimates and reduced MoCA scores at later time points (*β* = −0.12, *P* < 0.05), but not for chronological age (*β* = −0.10, *P* = 0.12). The significant effect of acute-phase brain age estimates remained significant (*β* = −0.42, *P* < 0.01) after adjusting for age, sex and education.

## Discussion

In this longitudinal study of working-age adults from two stroke cohorts, using a state-of-the-art brain age DL-based model on T1w MRI, we identified accelerated brain aging post-stroke, driven mainly by ipsilesional changes. Furthermore, we found a significant association between brain age estimates in the acute phase after stroke and long-term cognitive performance.

The observed accelerated brain aging in our two cohorts highlights the significant long-term impact of stroke on global brain health. Our findings of ipsilesional changes as the driver for accelerated brain aging post-stroke aligns well with a previous study using a cross-sectional design.^7^ There have been conflicting results for the trajectory of brain aging post-stroke.^6,32^ Our longitudinal data therefore corroborate the argument that stroke seems to accelerate brain aging, rather than being a static effect.

The negative correlation between within-subject longitudinal brain volume loss and increases in Brain-PAD suggest that the DL model captures biologically meaningful features related to brain atrophy. Further analyses using LME showed that accelerated brain aging over time was not fully explained by brain volume, indicating that there is more to brain aging than atrophy alone.

To enable comparison with previous post-stroke brain age studies using tabular data rather than raw MRI,^6,7,11,12^ we repeated our analyses using FreeSurfer-derived features and a ML model pre-trained on tabular data to estimate brain age. This analysis similarly demonstrated accelerated brain aging over time. In a separate analysis, we processed the MRI images with SynthSR for image standardization and lesion filling. While brain age estimates based on tabular data from the synthesized images continued to show accelerated brain aging, results from the DL model were not statistically significant and showed considerable variation, likely due to issues related to image intensity.

Our study aligns with and expands upon prior studies which have reported on cognition and brain aging post-stroke. Higher brain-PAD has previously been found to be associated with an increased risk of a post-stroke neurocognitive disorder,^33^ and increased Brain-PAD is associated with reduced performance on semantic and language tasks.^12^

Our study found that increased post-stroke brain age at admission correlates with reduced long-term cognitive performance, suggesting that brain age may be an early clinical prognostic biomarker, beyond chronological age. Importantly, these results were obtained using clinical MRI scans, demonstrating the feasibility of brain age as a clinically applicable biomarker that can be integrated into routine post-stroke assessment.

One limitation of our study is that the DL algorithm was applied without incorporating explainable AI, limiting the interpretability of the model’s estimations. However, it is worth noting that even when explainability is provided, it does not necessarily improve clinical decision-making.^34^ Layerwise relevance propagation has previously been implemented with the DL model investigating dementia and would be interesting to explore in a stroke cohort.^35^ Since the DL only takes whole-brain MRI as input, our analysis of ipsilesional and contralesional brain age estimations comes from mirrored hemispheres, which may have influenced the results.

Additionally, we performed a sub-analysis using FreeSurfer with the cross-sectional stream, ensuring that all scans underwent the same processing pipeline. While this approach maintains methodological consistency, using the longitudinal stream for patients with multiple scans could have yielded more accurate intra-individual estimates over time.

Another limitation is the risk of attrition bias due to loss to follow-up in cohort studies. In the CAST cohort, there were no significant differences between participants who completed the study and those lost to follow-up. In the OSCAR cohort, however, there were systematic differences in age and NIHSS, where younger patients and those with lower NIHSS scores were more likely to adhere, which could bias the results towards this subgroup.

We chose to focus this study on patients younger than 65 years at the time of stroke. This allowed for a more homogeneous sample with fewer age-related confounding brain pathologies, such as amyloid plaques, neurofibrillary tangles, microbleeds and small vessel disease. However, this age restriction inevitably reduces generalizability. Notably, our findings of accelerated brain aging after stroke are consistent with a previous study in a comparable age group (*N* = 47, mean age 57.7),^32^ whereas a study in an older population (*N* = 135, mean age 67.4) did not observe accelerated aging.^6^ As mentioned above, differences in methodology also need to be taken into account. Future studies should apply our methods to broader stroke populations, including older patients and those with haemorrhagic stroke.

Finally, the ceiling on lesion size and the high proportion of patients with large-vessel occlusion may limit the generalizability of the results. However, the comprehensive assessment of the cohort, the long-term follow-up with repeated measurements and the robustness of the findings should be considered important strengths of the study.

In conclusion, we identified accelerated brain aging up to 8 years post-stroke, challenging the notion of stroke as a purely acute event and highlighting the need for studies aimed at mitigating long-term neurodegeneration. The significant association between higher estimated brain age in the acute phase and reduced cognitive performance at follow-up strengthens the idea that brain age may serve as an early biomarker of post-stroke cognitive prognosis.

## Supplementary Material

fcaf509_Supplementary_Data

## Data Availability

Supporting data are available from the corresponding author upon reasonable request. All R codes used in the preparation of the manuscript are available through https://github.com/gislebh/Brain_age.
