# Supplementary material for: Brain age trajectories and cognition after stroke in two longitudinal cohorts
Source: Brain Commun. 2026 Jan 20;8(1):fcaf509. doi: 10.1093/braincomms/fcaf509 (PMC12816920; doi:10.1093/braincomms/fcaf509)
Supplement: fcaf509_Supplementary_Data [file fcaf509_supplementary_data.pdf]

Brain age trajectories and cognition after stroke in two longitudinal cohorts

Gisle Berg Helland, MD, Håkon Ihle-Hansen, MD, PhD, Anne Hege Aamodt, MD, PhD, Esten Høyland Leonardsen, PhD, Tobias Kaufmann, PhD, Brian Anthony B. Enriquez, MD, Mona K. Beyer, MD, PhD, Stein Andersson, PhD, Helle Stangeland, PhD, Hege Ihle-Hansen, MD, PhD, Hanne F. Harbo, MD, PhD, Einar August Høgestøl, MD, PhD*, Guri Hagberg, MD, PhD*

*Einar August Høgestøl and Guri Hagberg contributed equally to this work.

Supplementary Material

Supplementary Method:

Lesion segmentation

For patients in the OSCAR cohort, lesion segmentation was performed on diffusion-weighted imaging (DWI) acquired 24 hours after endovascular therapy, using ITK-SNAP.[1] The segmentation procedure has been described in detail in a previous publication.[2] In the CAST cohort, lesions were segmented using ITK-SNAP either on acute-phase DWI when available (n=22, 56%), or on FLAIR images acquired within the first-year post-stroke when acute DWI was not available (n=9, 23%). Stroke lesion volume (from this point referred to as lesion volume) was set to 0 for CAST patients with negative imaging findings and a clinical diagnosis of ischemic stroke (n=8, 21%). In both cohorts, lesion segmentation was performed by a trained researcher (G.B.H.) under the supervision of an experienced neuroradiologist (M.K.B.).

Environment and structure of the deep learning model

As the DL model is trained on heterogeneous datasets from multiple sites and scanners, generalizability is found to be good, and it has been shown to perform at state-of-the-art levels in both cross-sectional and longitudinal brain age estimation tasks.[3,4] The DL model was implemented in a Python 3.9 virtual environment created an managed with Miniconda (conda version 23.11.0)[5]. The DL model pipeline begins with skull stripping based on the FreeSurfer 5.3 autorecon pipeline,[6] followed by reorientation using FSL and registration to the MNI152 standard space using FSL's FLIRT tool.[7] The core of the DL model consists of a 3D Simple Fully Convolutional Network (SFCN) architecture with rectified linear unit (ReLU) activation

functions, six convolutional blocks, and a final regression node that outputs the estimated brain age.

Hemispheric mirroring

Each scan was split at the mid-sagittal plane, and one hemisphere (left or right) was mirrored using *numpy.flip* and concatenated with its original counterpart using *numpy.concatenate*.[8] This procedure produced two full-brain MRI volumes for each subject, one with the ipsilesional hemisphere mirrored and one with the contralesional hemisphere mirrored. Visual quality checks of the generated volumes were performed.

Freesurfer processing

To complement the deep learning estimations and improve model interpretability, we processed all T1-weighted images using FreeSurfer's cross-sectional recon-all pipeline (version 7.3.2).[6] The cortical and subcortical segmentations were subjected to visual quality control (QC), where it was seen that, as expected, patients with lesion volumes exceeding 70 ml had frequent distortions in surface reconstruction and cortical segmentation. This is a known problem in stroke neuroimaging studies.[9] Variable BrainSegVolNotVent is used as global brain volume, and eTIV is the measure used as intracranial volume (eTIV).

eXtreme Gradient Boosting

Features from FreeSurfer recon-all pipeline,[10] were extracted using the Human Brain Mapping atlas, providing tabular data which were then fed into an XGboost model, pre-trained on 1118 features from 35 474 individuals using several different public, collaborative an in-house cohorts, age spanning three to 89 years.[11] More details are publicly available at https://github.com/tobias-kaufmann/brainage.

SynthSR processing

SynthSR was applied to all NIfTI files in the study, including the 2D T1-weighted images acquired during the early stroke phase in the CAST cohort. The tool was run using FreeSurfer version 7.3.2, via the mri_synthsr command with the optional --lowfield flag enabled.[12] The output consisted of synthetic, lesion-filled, 1 mm isotropic 3D MPRAGE-like T1-weighted

images. These synthesized images were subsequently processed with both the DL model and the atlas-based XGBoost model.

Supplementary Results

Notably, the eXtreme Gradient Boosting model showed significant acceleration of brain aging after stroke, adjusted for sex and age, on both the native T1w images, and the SynthSR images ($\beta = 0.4$, $p < 0.01$ and $\beta = 0.5$, $p < 0.01$, respectively). Results from the DL brain age estimation on SynthSR images showed signs of deceleration of aging after stroke, but did not reach significance ($\beta = -0.6$, $p = 0.17$), with full results provided in Supplementary Table 3.

References:

1. Yushkevich PA, Yang G, Gerig G. ITK-SNAP: An interactive tool for semi-automatic segmentation of multi-modality biomedical images. *Annu Int Conf IEEE Eng Med Biol Soc*. 2016;2016:3342-3345. doi: 10.1109/EMBC.2016.7591443
2. Helland GB, Beyer MK, Enriquez BAB, Ihle-Hansen H, Ihle-Hansen H, Andersson S, Leonardsen EH, Stangeland H, Ùjhelyi B, Hagberg G, et al. Lesion Volume as a Predictor for Return to Work After Endovascular Treatment: A 4-Year Prospective Cohort Study. *Stroke: Vascular and Interventional Neurology*. 2024;4:e001494. doi: doi:10.1161/SVIN.124.001494
3. Dorfel RP, Arenas-Gomez JM, Fisher PM, Ganz M, Knudsen GM, Svensson JE, Plaven-Sigray P. Prediction of brain age using structural magnetic resonance imaging: A comparison of accuracy and test-retest reliability of publicly available software packages. *Hum Brain Mapp*. 2023;44:6139-6148. doi: 10.1002/hbm.26502
4. Skattebol L, Nygaard GO, Leonardsen EH, Kaufmann T, Moridi T, Stawiarz L, Ouellette R, Ineichen BV, Ferreira D, Muehlboeck JS, et al. Brain age in multiple sclerosis: a study with deep learning and traditional machine learning. *Brain Commun*. 2025;7:fcaf152. doi: 10.1093/braincomms/fcaf152
5. *Anaconda;* https://anaconda.com.: Anaconda Software Distribution; 2023.
6. Fischl B. FreeSurfer. *Neuroimage*. 2012;62:774-781. doi: 10.1016/j.neuroimage.2012.01.021
7. Jenkinson M, Beckmann CF, Behrens TE, Woolrich MW, Smith SM. Fsl. *Neuroimage*. 2012;62:782-790. doi: 10.1016/j.neuroimage.2011.09.015
8. Harris CR, Millman KJ, van der Walt SJ, Gommers R, Virtanen P, Cournapeau D, Wieser E, Taylor J, Berg S, Smith NJ, et al. Array programming with NumPy. *Nature*. 2020;585:357-362. doi: 10.1038/s41586-020-2649-2
9. Radwan AM, Emsell L, Blommaert J, Zhylka A, Kovacs S, Theys T, Sollmann N, Dupont P, Sunaert S. Virtual brain grafting: Enabling whole brain parcellation in the

presence of large lesions. *Neuroimage*. 2021;229:117731. doi: 10.1016/j.neuroimage.2021.117731
10. Fischl. FreeSurfer. *NeuroImage*. 2012;62. doi: 10.1016/j.neuroimage.2012.01.021
11. Kaufmann T, van der Meer D, Doan NT, Schwarz E, Lund MJ, Agartz I, Alnaes D, Barch DM, Baur-Streubel R, Bertolino A, et al. Common brain disorders are associated with heritable patterns of apparent aging of the brain. *Nat Neurosci*. 2019;22:1617-1623. doi: 10.1038/s41593-019-0471-7
12. Iglesias JE, Billot B, Balbastre Y, Magdamo C, Arnold SE, Das S, Edlow BL, Alexander DC, Golland P, Fischl B. SynthSR: A public AI tool to turn heterogeneous clinical brain scans into high-resolution T1-weighted images for 3D morphometry. *Sci Adv*. 2023;9:eadd3607. doi: 10.1126/sciadv.add3607

Supplementary Figures

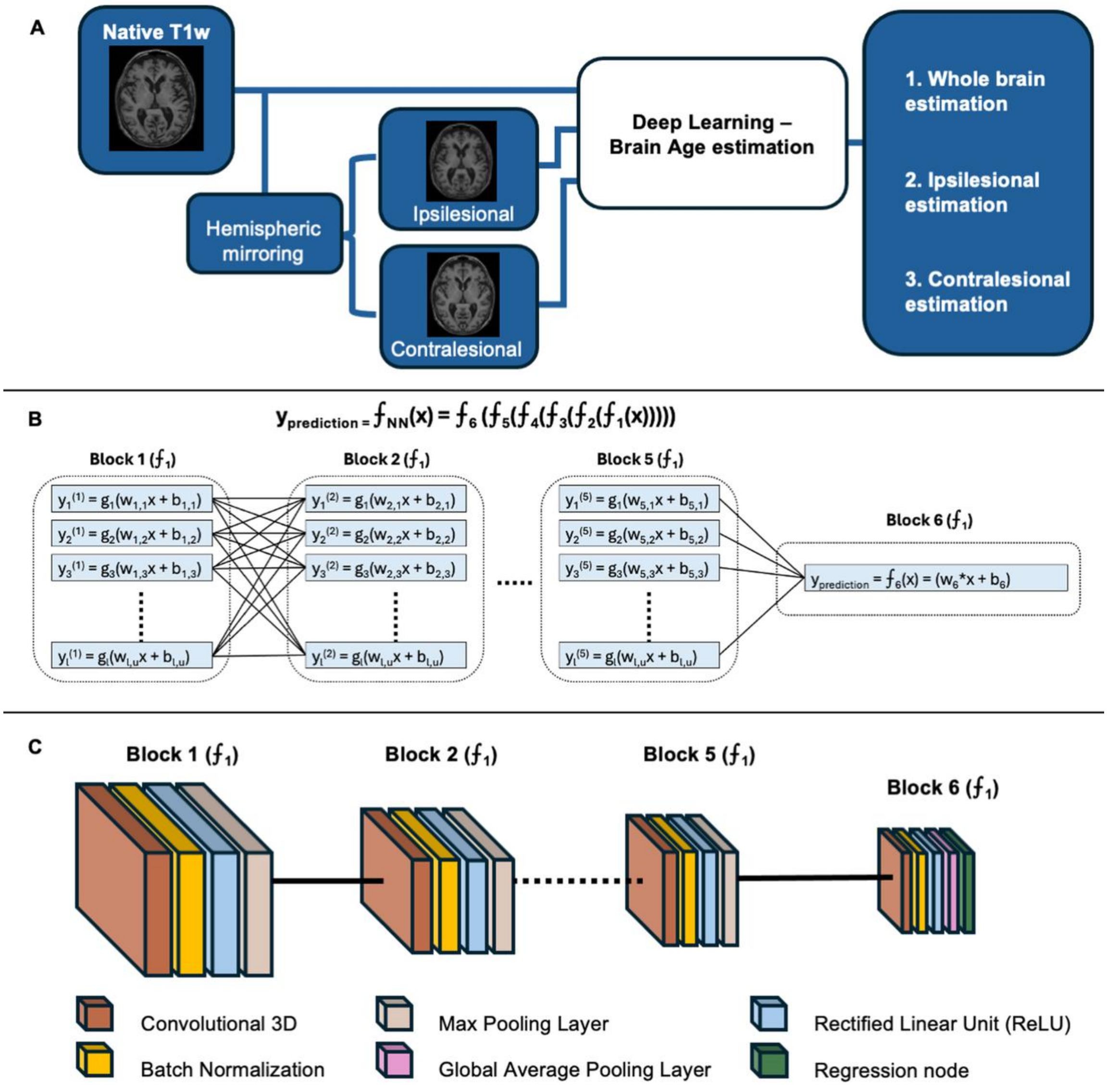

Supplementary Figure 1: Simplified overview of the brain age estimation workflow, resulting in three distinct outputs: a native whole-brain estimation, an ipsilesional estimation, and a contralesional estimation. A) Showing the flow of MRI images and creation of the ipsi- and contralesional images, ultimately resulting in three brain age estimations. B) Schematic and simplified mathematical visualization of the Pyment deep learning model. Each block is represented as a single layer composed of multiple units (neurons). L denotes the layer, u the unit, g the activation function, and w and b the learned weight matrix and bias vector, respectively. C) Conceptual overview of the Pyment model architecture. Each of the six blocks includes a sequence of operations: 3D convolution, batch normalization, rectified linear unit (ReLU) activation, and max pooling with spatial down sampling.

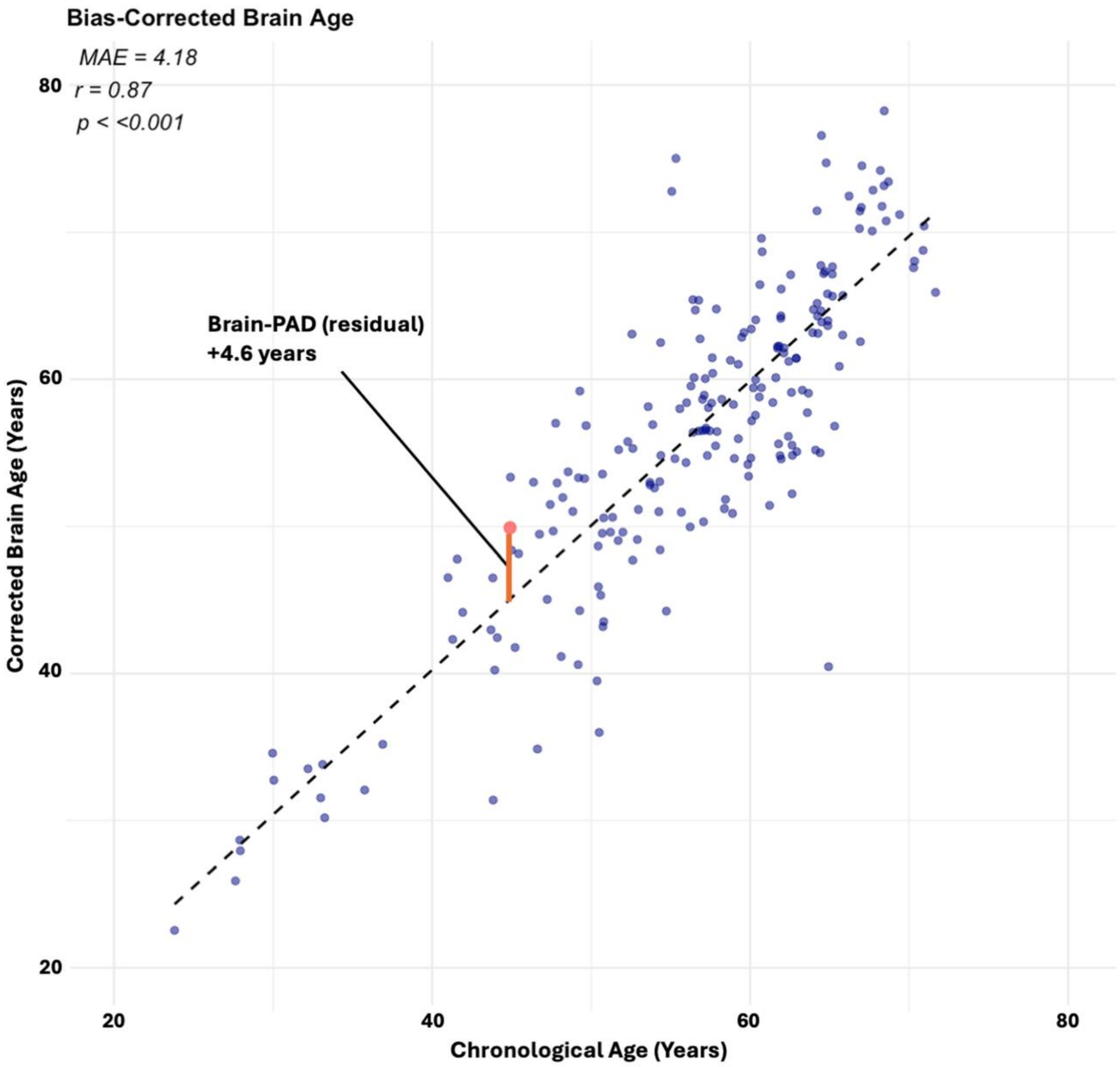

Supplementary Figure 2: Visualizing the Predicted Brain Age Difference (Brain-PAD), corrected for age and sex bias, for a single individual highlighted in red, N = 209. The Brain-PAD is shown as the residual (orange line), which is the vertical distance between the individual's predicted brain age and the expected value from the regression model. Other individual corrected Brain Age values are shown as blue dots. MAE; Mean Absolute Error, r; the Pearson correlation coefficient.

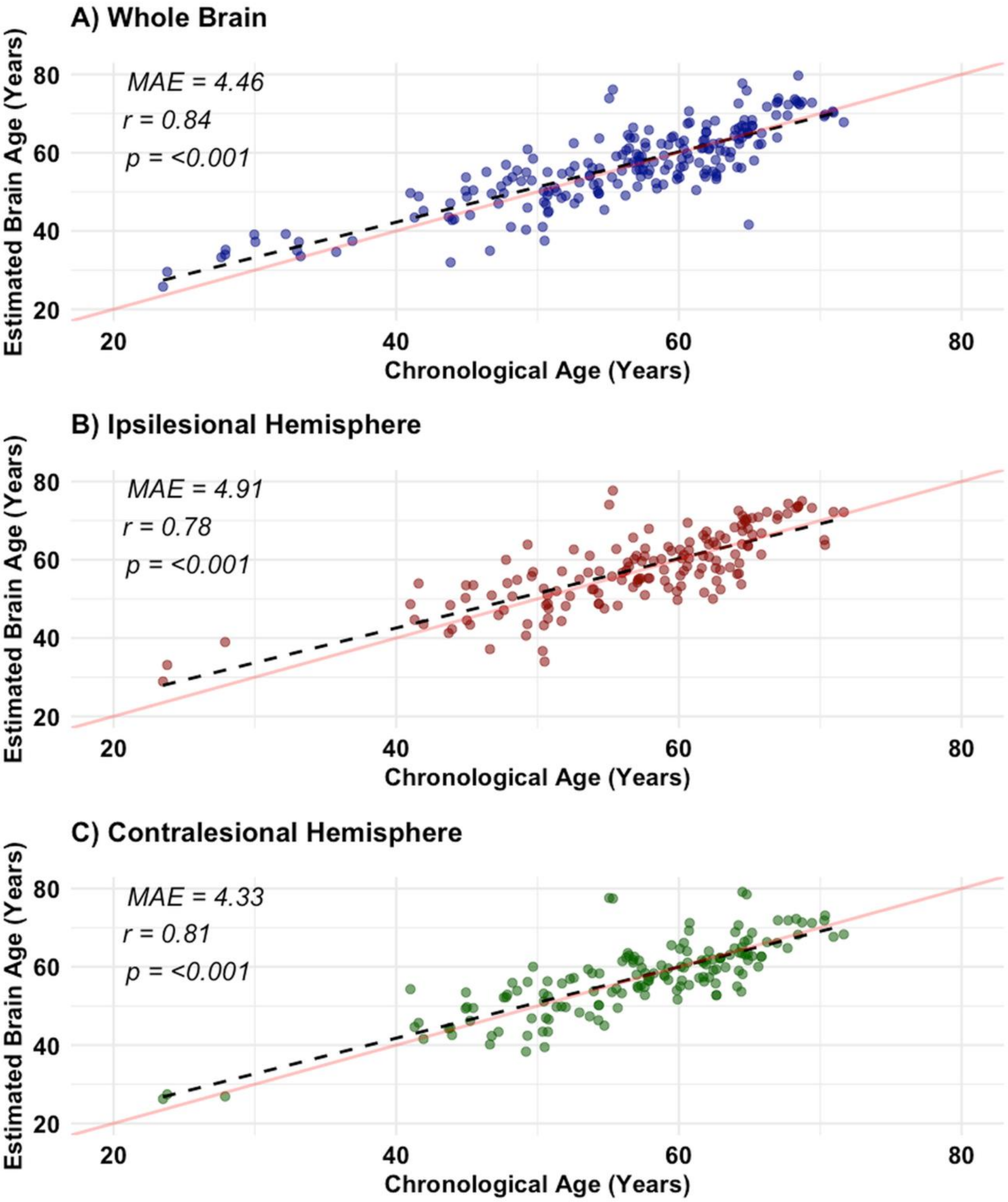

Supplementary Figure 3: Scatterplots of raw (uncorrected) deep learning estimated brain age versus chronological age, using the DL model on (A) whole brain N = 209 and (B) ipsi- and (C) contralesional hemispheres (both N = 155). Each colored dot represents a single scan. The red line represents the identity line (y = x), where predicted age equals chronological age. The black dashed line shows the mean across all data points.

Supplementary tables

Supplementary Table 1. The most common T1-weighted sequence parameters are reported by cohort. Slight deviations were observed, likely due to the clinical nature of the data, where radiographers may make minor adjustments to the scanning protocol.

OSCAR

T1w Parameter	Aera	Avanto	Skyra
**Acquisition type**	MPRAGE	MPRAGE	MPRAGE
**Slice thickness**	1.0	1.0	1.0
**spacing between slices**	0	0	0
**Voxel size, mm**	1.0 × 1.0 × 1.0	1.0 × 1.0 × 1.0	1.0 × 1.0 × 1.0
**Field -of -view, mm**	256 x 256	256 x 256	256 x 256
**Echo time, ms**	3	3	3
**Inversion time, ms**	900	900	900
**Flip angle, Degrees**	9	9	9

Most common parameter settings in scans for each scanner

CAST

T1w Parameter	First weeks after stroke	1 year after stroke	7 years after stroke
**Acquisition type**	Spin Echo	Gradient Echo	Gradient Echo
**Slice thickness**	5.0	1.0	1.0
**spacing between slices**	6.0	1.0	1.0
**Voxel size, mm**	0.9 × 0.9 × 5.0	0.9 × 0.9 × 1.0	0.9 × 0.9 × 1.0
**Field -of -view, mm**	240.0 × 240.0	240.0 × 240.0	220 × 220
**Echo time, ms**	14	4	4
**Inversion time, ms**	NA	2500	1660
**Flip angle, Degrees**	90	10	8

Most common parameter settings in scans for each timepoint.

Supplementary Table 2. Table of MR pipeline throughput and predictions

Session	1	2	3
**Patients, n**	120	100	73
**MR Scans, n**	103	87	67
**DL prediction – Native images, n**	92	80	64
**DL prediction – SynthSR images, n**	84	74	54
**DL prediction – Ipsilesional images, n**	64	58	45
**DL prediction – Contralesional images, n**	64	58	45
**Atlas-based prediction – Native images, n**	88	80	64
**Atlas-based prediction – SynthSR images, n**	71	67	43

All patients (n=120, had at least one MRI scan).

Supplementary Table 3: Demographic and clinical characteristics at admission and discharge for patients lost to follow-up at 2–14 months, compared with those who adhered to the study, including statistical comparisons between the groups. Results are presented separately for each original cohort.

Characteristics	Lost to follow-up at 3 months in OSCAR N = 20	Adhered to study at 3 months in OSCAR N = 62	Statistical comparison	Lost to follow-up at 12 months in CAST N = 0	Adhered to study at 12 months in CAST N = 38	Statistical comparison
**Admission**						
**Age,** in years, mean (SD)	**58 (8)**	**52 (10)**	***<0.05 †***	**-**	**58 (7)**	NA
**Sex,** female n (%)	**7 (35%)**	**29 (47%)**	**0.5‡**	**-**	**14 (37%)**	NA
**NIHSS,** mean ± SD; [range]	**15 ± 9 [2 - 39]**	**11 ± 7 [0 - 39]**	**0.1†**	**-**	**2 ± 2 [0 - 10]**	NA
**Arterial territory***			**0.8‡**			NA
**Left Media**	**9 (45%)**	**27 (44%)**		**-**	**11 (37%)**	
**Right Media**	**6 (30%)**	**23 (37%)**		**-**	**10 (33%)**	
**Posterior**	**5 (25%)**	**12 (30%)**		**-**	**9 (30%)**	
**Discharge**						
**Time since stroke,** days, mean ± SD; [range]	**1 ± 2 [0 - 5]**	**1 ± 1 [0 - 8]**	**0.8†**	**-**	**9 ± 6 [0 - 28]**	NA
**NIHSS,** mean ± SD; [range]^	**8 ± 10 [0 - 39]**	**4 ± 6 [0 - 39]**	**0.2†**	**-**	**1 ± 2 [0 - 10]**	NA
**Lesion volume,** mean ± SD; [range]	**23 ± 20 [0.1 - 68]**	**17 ± 15 [0.1 - 68]**	**0.4†**	**-**	**7 ± 14 [0 - 60]**	NA

OSCAR; Oslo Stroke Revascularization Study, CAST; Cognition After Stroke Study, SD; Standard Deviation, NIHSS; National Institutes of Health Stroke Scale. Missing; *8, ^1. Statistical test; †Wilcoxon rank-sum test, ‡Chi-squared test.

Supplementary Table 4: Demographic and clinical characteristics at admission and discharge for patients lost to follow-up at 3 - 7 years, compared with those who adhered to the study, including statistical comparisons between the groups. Results are presented separately for each original cohort.

Characteristics	Lost to follow-up at 3 years in OSCAR N = 36	Adhered to study at 3 years in OSCAR N = 46	Statistical comparison	Lost to follow-up at 7 years in CAST N = 11	Adhered to study at 7 years in CAST N = 27	Statistical comparison
**Admission**						
**Age,** in years, mean (SD)	55 (9)	52 (10)	0.3 †	57 (7)	59 (6)	0.6†
**Sex,** female n (%)	12 (33%)	24 (52%)	0.1‡	3 (27%)	11 (41%)	0.5‡
**NIHSS,** mean ± SD; [range]	13 ± 8 [1 - 39]	12 ± 8 [0 - 39]	0.6†	2 ± 2 [0 - 5]	2 ± 3 [0 - 10]	0.7†
**Arterial territory***			0.4‡			0.4‡
Left Media	19 (53%)	17 (53%)		5 (56%)	6 (29%)	
Right Media	11 (31%)	18 (31%)		2 (22%)	7 (33%)	
Posterior	6 (17%)	11 (24%)		2 (22%)	8 (3%)	
**Discharge**						
**Time since stroke,** days, mean ± SD; [range]	1 ± 1 [0 - 8]	1 ± 0 [0 - 3]	0.6†	7 ± 5 [0 - 19]	10 ± 7 [3 - 27]	0.1†
**NIHSS,** mean ± SD; [range]^	7 ± 8 [0 - 39]	4 ± 7 [0 - 39]	*<0.05†*	0 ± 1 [0 - 2]	1 ± 2 [0 - 10]	0.2†
**Lesion volume,** mean ± SD; [range]	20 ± 16 [1.6 - 68]	18 ± 17 [0.1 - 68]	0.3†	11 ± 16 [0 - 43]	6 ± 13 [0 - 60]	0.2†

OSCAR; Oslo Stroke Revascularization Study, CAST; Cognition After Stroke Study, SD; Standard Deviation, NIHSS; National Institutes of Health Stroke Scale. Missing; *8, ^1. Statistical test; †Wilcoxon rank-sum test, ‡Chi-squared test.

Supplementary Table 5. Results from Linear Mixed Effect models on prediction made by Model 1) Atlas-based XGBOOST model on native T1w images. Model 2) Atlas-based XGBOOST model on SynthSR T1w images. Model 3) Deep Learning model (Pyment) on SynthSR T1w images.

	Estimate	CI 2.5%	CI 97.5%	p-value
Model 1				
**Sex, female**	-0,03	-1,9	1,84	0,97
**Age**	0,03	-0,07	0,12	0,62
**Time Since Stroke, years**	0,4	0,11	0,68	**0,01**
**Lesion volume, log**	-0,68	-1,43	0,07	0,08
**Education, lower**	-0,29	-2,48	1,92	0,8
**Territory, posterior**	0,34	-2,17	2,84	0,79
**Territory, right**	-0,37	-2,59	1,86	0,75
Model 2				
**Sex, female**	-0,01	-2,01	1,98	0,99
**Age**	0,01	-0,1	0,12	0,82
**Time Since Stroke, years**	0,5	0,12	0,88	**0,01**
**Lesion volume, log**	0,12	-0,69	0,94	0,77
**Education, lower**	1,27	-1,24	3,8	0,33
**Territory, posterior**	-0,2	-2,82	2,41	0,88
**Territory, right**	0,81	-1,55	3,16	0,51
Model 3				
**Sex, female**	0	-3,58	3,58	1
**Age**	0	-0,19	0,19	1
**Time Since Stroke, years**	-0,55	-1,35	0,24	0,17
**Lesion volume, log**	-0,66	-2,12	0,79	0,37
**Education, lower**	-1,96	-6,7	2,79	0,42
**Territory, posterior**	-1,91	-6,72	2,9	0,44
**Territory, right**	-2,71	-6,91	1,5	0,21

Model 1: XGBoost model on FreeSurfer output model, from native images. Model 2: XGBoost model based on FreeSurfer output model from SynthSR images. Model 3: Deep learning model analyzing SynthSR T1w
